# 

**Published:** 1815-06

**Authors:** 


### MONTHLY CATALOGUE OF MEDICAL BOOKS.

THE Morbid Anatomy of the Liver:—Part 2, on the Varieties of the Tubera Diffusa. By J. R. Farre, M.D. 4to.—Longman and Co.

The History of the Small-pox. By James Moore. 8vo.—Longman and Co.

Memoirs of Military Surgery. By D. J. Larrey, M.D. Abridged and translated from the French; by John Waller, Surgeon of the Royal Navy. Part 1. 8vo.—Cox and Son.

A Practical Inquiry into Disordered Respiration, distinguishing the Species of Convulsive Asthma, their Causes, and Indications of Cure. By Robert Bree, M.D. F.R.S. Fellow of the Royal College of Physicians. The fifth edition, corrected and improved, with additional observations. 8vo.—Callow.

Reflections on Fever, intended to point out the Principles upon which a systematic and useful Method of Treatment might be established. By Robert Calvert, M. D. Physician to the Forces, &c. &c. &c. 8vo.—Callow.

Additional Reports on the Effects of a peculiar Regimen in Cases of Cancer, Scrofula, Consumption, Asthma, and other Chronic Diseases. By William Lamb, M.D. 8vo.—Mawman.

Researches on Pulmonary Phthisis. From the French of G. L. Bayle; by William Barrow, M.D. 8vo.—Longman and Co.

A View of the Relations of the Nervous System in Health and in Disease; containing Selections from a Dissertation to which was adjudged the Jacksonian Prize for the year 1818, with additional Illustrations and Remarks. By Daniel Pring, Member of the Royal College of Surgeons, London, and Surgeon at Bath. 8vo.—Callow.

---

### NOTICES TO CORRESPONDENTS.

*Our correspondent in America advises us that he has forwarded the American Journals by a vessel bound to Liverpool. Our letters are by a private hand; but, from some delay at the Custom house, the packet has not arrived. We promise ourselves much information from it, as our transatlantic brethren have been very busy in repeating experiments reported in the transactions of the various societies of Great Britain.*

*Communications are received from Drs. Kinglake, Walker, Brown, and various anonymous Correspondents; and several new publications from their respective authors. These shall be attended to in their order.*

### INDEX

### TO THE THIRTY-THIRD VOLUME.

ACID, chromic, on some unknown combinations of 33
Adams, Dr. Jos. on Goître and Cretinism 281, 433
Affusion, cold, effects of, in typhus and scarlatina 359
Amaurosis, case of, successfully treated by electricity 468
ANALYSIS, CRITICAL, of new publications 49, 128, 221, 315, 406, 485
Anatomie et Physiologie du Systeme nerveux, &c. par F. J. Gall & G. Spurzheim, &c. critical analysis of 228
Aneurism, popliteal, description of Mr. Crampton's operation for 158
——— of the aorta, case of, with peculiar symptoms 265
———, subclavian, case of, terminating favourably without an operation 377
Aorta, obstructed, case of 506
Apothecaries, statement of their design in their Bill 423
Argenti nitras, effects of, in chorea sancti viti 273
Atkinson, Mr. T. his case of puerperal peritonitis 447
Bateman, Dr., remarks on his description of elephantiasis 107
Bellingham, Mr. R., his case of hydrophobia 13
Blumenbach, his Institutions of Physiology 239
Bone, pieces of, discharged from ulcers in the sternum 8
Books publishing and published 74, 168, 252, 344, 432, 516, 520
Botanology, Welsh, critical analysis of 409
Brain, case of abscess in the 60
——, morbid anatomy of, in mania and hydrophobia 139
Broussais, M., his account of eight tape-worms existing at the same time in one individual 7
Bryant, Mr. T., his case of Ann Ferry, with the dissection 358
Bush, Mr. F. on fractures of the extremities 170
Buxton, Dr. on the effects of temperature in consumptive disorders 365
Cæsarian operation, account of its being successfully performed 162
Cagots, history of 210
Carpue, Mr., account of his Taliacotian operation 160
CASES—of tape-worms 7
——— pieces of bone discharged from the sternum 8
——— hæmorrhagic disposition in several individuals of one family 9, 12
——— hydrophobia 12, 16
——— pneumonic inflammation 28, 30, 171, 233
——— diseases of the heart 49, 72
——— abscess in the brain 60
——— laceration of the internal coat of the stomach and duodenum 64
——— extraordinary detention of a fœtus in the body of the mother 65, 147
——— of a white man becoming black 73
——— a brainless child 152
——— the Cæsarian operation 162
——— necrosis 190, 512
——— recovery from opium 201
——— swallowing laudanum 201, 350
——— pulmonary disease 207
——— deafness 227
——— diseased rectum 237
——— gout 264
——— aneurism of the aorta 265
——— chorea sancti viti 273
——— supposed confluent varicella 274
——— black cataract 312
——— a mass like a placenta discharged from the womb, without a fœtus 333

CASES—of hydatids and a placenta brought away without a fœtus 346
———— ruptured uterus 359
———— subclavian aneurism 377
———— muscæ volitantes 412
———— puerperal peritonitis 447
———— ptyalism 456
———— amaurosis 468
———— injury of the hip 469
———— obstructed aorta 506
———— preternatural labour 514
Cataract, black, observations on 311
Cerebellum, state of, in epileptics 182
Cheston, Dr. R. B., his account of a child retained in the mother fifty-two years after the usual period 65, 147
Chevalier, Mr, his case of laceration of the internal coat of the stomach and duodenum 64
China, state of medicine in 247
Clarke, Dr., his annual meteorological table for Sidmouth 188
Coffin, Dr. J. G., his cases of ruptured uterus 359
Colchicum, tincture of, its effects in gout 264
Cold, some peculiar effects of 246
COLLECTANEA MEDICA 33, 108, 209, 298, 391, 471
Collins, Dr. J. C., his case of hydatids and placenta without a fœtus 346
Consumption, effects of temperature in 302, 365
Copeland, Mr. T., on diseases of the rectum 237
————, on the symptoms and treatment of diseased spine 406
Cradle, elastic iron, for fracture, described 170
Crampton, Mr., account of his operation for popliteal aneurism 158
Cretinism, remarks on, by Dr. Adams 433
Croup, historical fragment concerning 247
Crowfoot, Mr., his case of amaurosis 468
Davies, Mr. Hugh, on Welsh Botanology 409
Davy, Dr. J., experiments of, on animal heat 113
——, Sir H., on iodine 37
————, experiments of, on the combustion of the diamond 121
———————— other carbonaceous substances 125
Deafness, case of 227
Denmark, Dr., his case of abscess in the brain 60
Dew, essay on, by Dr. Wells, critical analysis of 232
Digestion, review of Dr. de Montegre's experiments upon 420
Diseases, monthly report of 167, 254, 341, 431, 519
Dissections 7, 31, 66, 99, 140, 147, 199, 222, 270, 317, 358, 362, 506
Druggists, on the evil practices of some 101
Drugs, London prices of 76, 256, 342, 429, 517
Edinburgh Medical and Surgical Journal, critical analysis of 145, 221, 332
Egypt, on the climate and pyramids of 392
Electrical phenomena, account of some 249
Elephantiasis, remarks on Dr. Bateman's description of 107
Epidemic in New York, account of an 92
Epileptics, state of the cerebellum in 182
Epizootic near Paris, account of an 160
Farre, Dr. J. R., on malformations of the human heart 49
Ferry, Ann, case of, and dissection 358
Fever, yellow, remarks on the treatment of 5
——, puerperal, effects of oil of turpentine in 180
——, epidemic, at Gibraltar, in 1804, 1810, and 1813, account of 424
Franklyn, Mr. F. E., on the use of argenti nitras, combined with the shower bath, in chorea 273
Fungus cerebri, on the treatment of 403
Gall, Dr., remarks on his Physiognomical System 485
Gamage, Dr. W. jun., his cases illustrating the efficacy of bleeding 28
Garat, M., his account of the pyramids of Egypt 392
Des Genettes, Baron, his exposé of the state of medicine in France in 1814 298
Gilpin, Dr. J. D. A., on an epidemic fever at Gibraltar 424
Goîtres, account of 209, 433
Gout, remarks on the nature, and means of prevention, of 2, 4
——, effects of colchicum in 264
Graham, Dr., his case of obstructed aorta 506
M'Gregor, Mr. P., remarks on his Report of the Diseases of the Children of the Military Asylum at Chelsea 418
Guy, Mr. W., on the treatment of rabies contagiosa 293
Harvey, Dr., memoirs of the celebrated 108

Hay, Dr. John, his account of a hæmorrhagic disposition in many individuals of the same family 9, 12
Haxby, Dr., his case and dissection of aneurism of the aorta, with remarkable appearances 265
———, his reply to the editors 449
Heart, on malformations of, by Dr. Farre 49
——, congenital hernia of the, case of 72
Heat, animal, experiments on 113
Hip, injury of, confounded with dislocation, and fracture of the neck of the thigh bone 469
Hume, Mr., remarks on his paper on barytes 35
———, his answer to ditto 248
———, his improved method of making antimonium tartarizatum 484
Hunterian oration, notice of the 245
Hydrophobia, case of, in which bleeding was unsuccessful 13
———, dissection of a case of 140, 317
———, on blood-letting in 178
———, suggestions relative to the treatment of 293
Inflammation, pulmonic remarks on, with cases 171
INTELLIGENCE, Medical and Philosophical 69, 157, 243, 336, 422, 514
Institute, French, proceedings of 396
Iodine, experiments and observations upon 37
Itch, remedy for 246, 515
Jackson, Dr. J. his case 27
James, Mr., on the nervous system 198
John, Professor, on some unknown combinations of chromic acid 33
Jones, Mr. R., on the effects of large quantities of laudanum, with cases 350
Key, Mr. on blood-letting in hydrophobia 178
Kinglake, Dr. on the effects of oil of turpentine in puerperal fever 179
———, on vaccine and variolous inoculation 257
———, on general and local disease 459
Labour, preternatural, case of 514
Lactuca *virosa* recommended as a remedy for pertussis 161
Lawrence, Mr. his case of a child born without a brain 158
Laudanum, cases in which large quantities were taken 201, 350
LECTURES announced.—On Medicine—Dr. Hooper and Dr. Ager 74, 163
LECTURES announced.—On Medicine—Dr. Babington and Dr. Curry, Dr. Adams 75
——— Dr. Clutterbuck, Dr. Roget 429
——— On Anatomy, Surgery, and Physiology—Mr. Brookes 74
——— Mr. A. Cooper and Mr. H. Cline, Dr. Haighton, Mr. Taunton 75, 429
——— Mr. Pettigrew 253
——— Mr. Shaw 429
——— On Midwifery and Diseases of Women and Children—Dr. Haighton, Dr. Squire, Dr. Clarke and Mr. Clarke, Dr. Merriman, Dr. Clough 75, 253, 429
——— On Chemistry, Botany, and Materia Medica—Dr. Curry and Dr. Cholmeley, Mr. Singer 75
——— Dr. Hooper and Dr. Ager 163
——— Dr. Clutterbuck, Dr. Harrison 429
——— On Experimental Philosophy, Mr. Allen 75
——— On the Structure and Diseases of the Teeth—Mr. Fox ib.
——— Mr. Nicholas 163
Lemon, Mr., his case of placenta discharged without a fœtus 333
M'Leod, Mr. J., on the treatment of yellow fever 5
Lignum, Mr. J., on tincture of colchicum in gout 264
Lodge, Mr. T., on cold affusion in typhus and scarlatina 359
Machell, Mr. his description of an annular saw 189
———, his case of necrosis 190
Marshal, Dr. A., his life and works 54, 139
Matthias, Mr. P., his case of Joanna Southcott 139
Medico-chirurgical Transactions, vol. v. critical analysis of 60, 147, 410, 506
Medicine, state of, in China 248
——— France in the year 1814 298
Mesenteric glands, diseased, remarks on 291
Meteorological register 79, 164, 255, 343, 430, 518
——— table for Sidmouth, Devon 188
Mitchell, Dr. J., his case of pieces of bone discharged from ulcers in the sternum 8
De Montegre, Dr. A. J. on digestion 420

Monthly catalogue of medical books 80, 168, 256, 344, 432, 520
——- prices of drugs 77
——- report of diseases 166, 254, 341, 431, 519
Mortality, bill of, for 1814 165
Moxa, application of, to the head, successful in a case of inflammatory catarrhal head-ach 426
Muscæ volitantes, case of 412
National Vaccine Establishment, proceedings of 69
Natural history, some interesting particulars of 339
Necrosis, case of 190
——- of the tibia, observations on 510
————, case of 512
Nervous system, on the functions of 23, 82, 276, 351, 456
————, its importance 198
Notices to correspondents and readers 80, 168, 256, 344, 432, 520
Opium, case of recovery from 201
Orfila, M. Report to the French Institute of his Toxicologie Generale 471
Parry, Dr. C. H., on tetanus and hydrophobia 315
Peritonitis, puerperal, case of 448
Phillips, Mr. R., on Mr. Hume's paper on barytes 35
————, answer to him by Mr. Hume 248
Physiognomical System of Gall and Spurzheim, critical analysis of 485
Physiology, Institutions of, by Blumenbach, critical analysis of 239
Plague at Malta, account of the 477
Plates 81, 169, 433
Poisons, experiments relating to 472
Porrigo, treatise upon, by the late Dr. Willan 128
Prices of drugs 76
——- of substances used in pharmacy 77
Proprietors, their address to the public 1, 345
Pseudo-syphilis, remarks on 193
Ptyalism, observations on 454
——-, cases of 456
Purgatives, drastic, impropriety of administering in certain worm-cases 292
Quackery, observations upon 21
Queries addressed to Mr. Smerdon 187
Rectum, Diseases of, critical analysis of 236
——-, case of 237
Reece, Dr. R., on the illness and death of Mrs. Southcott 137
Rees, Mr. Leyson, on the case of W. O. 81
Report of diseases 167, 254, 341, 431, 519
——-, official, to the French Institute on M. Orfila's "Toxicologie Generale" 471
——- of the General Committee of Associated Apothecaries 157
——- of the Hospital for Small-pox Inoculation and Vaccination 250
——- of the Natural Diseases of the Children of the Military Asylum at Chelsea, critical analysis of 418
——-, special, of the General Committee of the London Infirmary for Curing Diseases of the Eye, critical analysis of 327
——- of the City or Royal Hospitals 340
Robertson, Dr. W., on pulmonic inflammation 171, 283
————, on ptyalism, with cases 454
Rowe, Mr., his case of recovery from opium 201
——-, his case of supposed confluent varicella 274
Saw, annular, description of 189
Sawrey, Mr. S., his life and works of Dr. Marshal 54, 139
Skinner, Mr. J., on the late plague at Malta 477
Small-pox, observations on 258
Smerdon, Mr. C. W. on the functions of the nervous system 23, 82, 276, 351, 450
Smith, Ashby, his edition of Dr. Willan's treatise on Porrigo, &c. 128
Society, London Medical, proceedings of 71, 336
——-, Medical and Chirurgical, of London, officers and council, 1815 422
——-, Medico-Chirurgical, of Gand, proceedings of 71
——-, Royal, of London, ditto 157, 243
————, of Edinburgh 244, 514
——-, London Truss, account of the 245
Southcott, Joanna, particulars of her case 137
Spine, diseased, on the treatment and symptoms of 406
Spurzheim, Dr. his Physiognomical System, critical analysis of 485
Starr, Mr. R. N., his observations on the case of W. O. 2
Stevens, Mr. G., on quackery 20

Stevenson, Mr., on the operation for artificial pupil 192
————, his case of deafness 226
Subclavian artery, operation for tying, unsuccessful 221
Sutton, Dr., on high temperature in consumption 202
Syphilis, remarks upon 262
T., Lieut.-Col., singular case of 264
Tape-worms, account of eight in one individual at the same time 7
Tennant, Smithson, Esq., particulars of his death 428
Tetanus, observations on 316
Travers, Mr., remarks on his observations on cataract 416
Truss, description of a new-invented spring 104
Turpentine, oil of, its effects in puerperal fever 179, 448
Uranolytes, fall of, near Agen 215
Urine, description of some that was milk-white 160
Uterus, ruptured, cases of 359
Vaccination, remarks upon 18, 257
Vænesection, utility of, in certain cases 27
————, on the effects of, in hydrophobia 178
Vaughan, Dr., his letter to the editors 345
Walker, Dr. J. K., on the danger of mistaking certain infantile affections for worm-cases 290
Walker, Mr. R., on gout 2, 4
————, his reply to Mr. Woodham 16, 253
————, on vaccination 18
————, on pseudo-syphilis 193
————, on wounds and ulcers 383, 462
Ware, James, Esq., on muscæ volitantes 412
————, his death announced 427
Wells, Dr., his Essay on Dew 233
Wenzel, Professor, on the state of the cerebellum in epileptics 182
————, John and Charles, on the treatment of fungus cerebri 403
Whately, Mr. T., his Observations on Necrosis, critical analysis of 510
Whitford, Mr. J., his new spring truss 104
Willan, Dr., on porrigo and impetigo 129
Wilson, Mr. J., on the case of W. O. 262
W. O., remarks on the case of 2, 81, 193, 261
————, reply to the inquiries of 32
Woodham, Mr., his objections to some opinions of Mr. Walker 89
Yeatman, Mr. J. C., his case of subclavian aneurism 377
————, ditto of injury of the hip 469

### PLATES.

J. Whitford's Patent Truss - - - - - Page 81
Mr. F. Bush's Elastic Iron Cradle for Fracture of the Leg - 169
Diagrams to illustrate Dr. Spurzheim's Treatise - - 433

